# Supplementary material for: Increased cancer risk in HIV-infected individuals occupationally exposed to chemicals: Depression of p53 as the key driver
Source: PLOS Glob Public Health. 2024 Jul 23;4(7):e0002841. doi: 10.1371/journal.pgph.0002841 (PMC11265661; doi:10.1371/journal.pgph.0002841)
Supplement: Attachment — Submitted filename: Response to Reviewers.docx [file pgph.0002841.s001.docx]

**April 17, 2024**

The Editor in Chief

PLOS Global Public Health

Dear Sir/Madam,

**Re: Revision required [PGPH-D-23-02537] - [EMID:4e7fcbba09534b12]**

We have revised our manuscript titled "Occupational cancer risk surveillance in HIV-infected individuals exposed to chemicals: Role of p53 molecular marker," with ID PGPH-D-23-02537. This update is to provide information about the revisions.

In this revision, we carefully addressed each comment and suggestion from the reviewer to enhance the quality and clarity of our work. Please refer to the next page for details of the revisions made. Line numbers referenced in the authors' response column directly correspond to line numbers in the file 'Revised Manuscript with Track Changes.'

We would like to express our gratitude to the reviewer for providing constructive feedback and valuable input that have strengthened the manuscript.

Yours faithfully,

Donald C. Udah

| **SN** | **Comments** | **Author’s response** |
| --- | --- | --- |
| **Journal Requirements:** | | |
| 1 | Please ensure that Funding Information and Financial Disclosure Statement are matched. | I have deleted the financial disclosure statement and other statements related to author contributions from my manuscript file, in compliance with the journal's requirement. I correctly entered the Financial Disclosure Statement in the Financial Disclosure section of the submission form. Contributions for each author were already entered in the submission system. |
| 2 | In the Funding Information you indicated that no funding was received. Please revise the Funding Information field to reflect funding received. | **No funding was received.** The line (before line 480) containing the text “Funding acquisition: Donald C. Udah, John I. Anetor” has been deleted. |
| **Response Reviewers' comments:** | | |
| 3 | The title of the manuscript did not reflect the entirety of the work done. There should be a way to ensure that the entirety of the work done is captured to give correct first-sight impression about the study. | **Rectified.** The title of the manuscript has been revised from ‘Occupational cancer risk surveillance in HIV-infected individuals exposed to chemicals: Role of p53 molecular marker’ to ‘Increased cancer risk in HIV-infected individuals occupationally exposed to chemicals: depression of p53 as the key driver’, See lines 2-4. |
| 4 | Line 34: The use of the word “Apparently healthy adults” to include HIV patients may be inappropriate. The statement should read “Study Participants or Subjects…). | The reviewer’s suggestion incorporated. The phrase “Apparently healthy adults” on line 37 has been changed to “Study participants” |
| 5 | Line 35: bcl2 should be written in full, followed by being put in bracket for subsequent use | **Rectified.** β-cell lymphoma-2 (bcl2) has been written in full at the first mention on line 40. |
| 6 | Lines 65-66: “..such as Kaposi sarcoma and non-Hodking’s lymphoma” can be moved to line 61, after “ AIDS -defining cancers…” to actually define the AIDS-defining cancers are. | **Done.** Lines 64 and 69 have been updated to reflect the reviewer's recommendation. |
| 7 | Line 101: the interplay of that of bcl2 was not well elucidated like that of p53. | **Rectified**. Lines 107-111 have been added to provide further context for bcl2. |
| 8 | Line 107: It will be great if the description of “the community” and the “the health facility” can be stated, to define the study site. | **Done.** Study site has been described on lines 121 and 123. |
| 9 | Line 111: ‘and’ all recruited into the study | **Done.** Line 127 has been updated to reflect the reviewer's recommendation. |
| 10 | Line 113: replace “elicit” with “obtain” | **Done.** Line 128 has been updated to reflect the reviewer's recommendation; “elicit” has been replaced with “obtain”. |
| 11 | Lines 120 – 123: It will be good if the nature of toxicants that the subjects were exposed to, based on their occupations, are mentioned here, then supported by the references. | **Done.** The reviewer's recommendation has been addressed on lines 138-141: “Therefore, the occupationally exposed HIV-infected and uninfected participants were exposed to toxicants including automotive chemicals, cement dust, diesel exhaust, electronic waste, fungicides and pesticides, metal cutting fluids, metal fumes, secondhand smoke, solvents, sulfuric acid, and wood dust.” |
| 12 | Lines 127 - 132: Please reframe this paragraph. Perhaps you start with “participants with history of hereditary …… were excluded from this study”. | **Done.** The reviewer's recommendation has been incorporated on lines 144-148: “Participants with the history of hereditary immune deficiency, primary immune deficiency, autoimmune disease, immunosuppressive drug use, previous cancer diagnosis, supplement use at the time of the study or within the month preceding it, refusal to consent, or age <18 years were excluded from this study.” |
| 13 | Line 138: Please state the quality control measures taken, to achieve accurate anthropometric measurements. | **Rectified.** The reviewer's recommendation has been incorporated on lines 157-163: “The weighing scale and stadiometers were calibrated with standard weights and known height measurements respectively. Participants were instructed to take off their shoes, coats, and hats (if they were wearing them). The participants' heights were measured while they stood straight against the stadiometer, with their heels, buttocks, shoulders, and heads touching the vertical surface. During weight measurement, participants were instructed to remain still in the centre of the weighing scale.” |
| 14 | Line 147: Please be specific with the centrifugation process. The centrifuge speed cannot be ranged. | **Rectified.** Centrifugation speed has been changed from ‘2200-2500 RPM’ to ‘2500 revolutions per minute (rpm)’ in line 169. |
| 15 | Line 153-154: please reframe the statement. It is not explicit enough | **Rectified.** The statement has been reframed: “The Becton Dickinson (BD) FASCount system (Becton, Dickinson and Company, California, USA) was used to identify and count CD4+ T cells. This flow cytometry method employs specific monoclonal antibody and fluorescence-activated cell sorter (FACS) analysis”. Additionally, further context has been provided to improve clarity. See lines 182-193. |
| 16 | Table 1 refers: It will be better if another column is created to express the tools of the numerical variables (the t statistic). It is not actually clear with the way it is presently expressed. | **Done.** The reviewer's recommendation has been addressed: The numerical demographic information has been moved below the categorical variables for better organization. Additionally, superscripts have been added to the continuous variables and the t-statistic, along with explanatory footnotes, in order to enhance clarity. |
| 17 | Lines 203 -205: Seem tautologic. Remove and report the BMI outcome between HIV positive and Negative | **Rectified.** The BMI outcome between HIV-positive and HIV-negative in the unexposed category has been properly reported. See line 255 for correction. |
| 18 | Lines 206 – 208: Should preceded the sociodemographic descriptions in line 197. | **Rectified.** The text, “The participants' ages ranged from 18–65 years, with a mean ± SE of 38.35 ± 0.72, and they were found to have worked for an average of 10.57 ± 0.61 years. The mean duration of HIV exposure in the HIV positive group was 42.67 ± 2.45 months.”, has been moved to the recommended position. See lines 243-246. |
| 19 | Figure 2 refers: The figures did not adequately display the proper interpretation of the outcomes. Only the F-value could be observed. The bars showing individual comparisons within and between groups (post-hoc) are not displayed. This should be corrected. All labelled bars should be properly annotated. | **Rectified.** Figure 2 has been appropriately labelled and uploaded. Clear explanatory notes have been included as figure captions in the text of the manuscript to aid in proper interpretation (lines 300-311): “Fig 2. Levels of p53 and other markers in HIV-negative unexposed (HNU), HIV-negative exposed (HNE), HIV-positive unexposed (HPU), and HIV-positive exposed (HPE) groups.  Bar graphs display the mean values of biomarkers p53, β-cell lymphoma (bcl2), 8-hydroxydeoxyguanosine (8-OHdG), superoxide dismutase (SOD), malondialdehyde (MDA), and CD4 counts, respectively, across study groups, with error bars indicating the standard error of the mean. In each plot, the bars are arranged in descending order of mean values for quick identification of trends in the data and clearer comparison between the different categories represented by the bars. Additionally, the overall p-value across the study group derived from a one-way ANOVA is indicated at the top of each figure. Pairwise comparison results (post hoc analysis) are represented by letters positioned above bars: When two bars do not share a common letter, it indicates a significant difference between study groups represented by the bars (p < 0.05).”  Additionally, Fig 2 was uploaded to PACE and refined to meet the requirements set by PLOS. |
| 20 | Line 256: The said bar chart in Figure 2 could not be found. I am of the opinion that subsequent tables and figures are displayed separately from Figure 2. Missing figures could be clearly seen because of the much figures expressed already, with no distinct referral labels to them. | **Rectified.** To be clear and concise, texts mentioning the missing figure have been removed. |
| 21 | Table 3 : The title “Immunological Status” is a broad statement. It could be put as “Immunological Status of Study Groups based on the CD4 Levels”. | **Rectified.** Table 3 title has been adjusted to: “Immunological status of study groups based on CD4 counts”. See line 344. |
| 22 | Line 299: “compared to” instead of “in comparison to” | **Done.** The reviewer's recommendation has been incorporated; the phrase “in comparison to” has been replaced with “compared to”. See line 389. |
| 23 | Line 322: The first statement required a reference. | Done. A reference has been added (ref. 44). |
| 24 | Line 362: Replace the word “prior” with “previous” | **Done.** The reviewer's recommendation has been incorporated; the phrase “prior” has been replaced with “previous”. See line 453. |
