# Supplementary material for: Increased cancer risk in HIV-infected individuals occupationally exposed to chemicals: Depression of p53 as the key driver
Source: PLOS Glob Public Health. 2024 Jul 23;4(7):e0002841. doi: 10.1371/journal.pgph.0002841 (PMC11265661; doi:10.1371/journal.pgph.0002841)
Supplement: Attachment — Submitted filename: Response to Reviewers.docx [file pgph.0002841.s003.docx]

**June 11, 2024**

The Editor in Chief

PLOS Global Public Health

Dear Sir/Madam,

**Re: Revision required [PGPH-D-23-02537R1]**

Thank you for your feedback regarding our revised manuscript titled "Increased cancer risk in HIV-infected individuals occupationally exposed to chemicals: depression of p53 as the key driver," with ID PGPH-D-23-02537R1.

We have revised our manuscript carefully, addressing each comment and suggestion from the reviewer. Please refer to the next page for details of the revisions made. Line numbers referenced in the authors' response column directly correspond to line numbers in the file 'Revised Manuscript with Track Changes.'

Thank you once again for your thorough review, attention to detail and valuable inputs.

Yours faithfully,

Donald C. Udah

| **SN** | **Comments** | **Author’s response** |
| --- | --- | --- |
| **Journal Requirements:** | | |
| 1 | Please review your reference list to ensure that it is complete and correct. If you have cited papers that have been retracted, please include the rationale for doing so in the manuscript text, or remove these references and replace them with relevant current references. Any changes to the reference list should be mentioned in the rebuttal letter that accompanies your revised manuscript. If you need to cite a retracted article, indicate the article’s retracted status in the References list and also include a citation and full reference for the retraction notice. | A. Reference No. 4 has been updated: The missing volume, issue, and page numbers have been added. This reference was changed from “4. Castelli R, Schiavon R, Preti C, Ferraris L. Hiv-Related Lymphoprolipherative Diseases In The Era Of Combination Antiretroviral Therapy. Cardiovascular & hematological disorders drug targets. United Arab Emirates; 2020. doi:10.2174/1871529X20666200415121009” to “Castelli R, Schiavon R, Preti C, Ferraris L. HIV-Related Lymphoprolipherative Diseases In The Era Of Combination Antiretroviral Therapy. Cardiovascular & Hematological Disorders Drug Targets. 2020. pp. 175–180. doi:10.2174/1871529X20666200415121009”. B. Reference No. 9 has been updated: The missing DOI ‘10.1155/2019/3030614’ was included. |
| **Response Reviewers' comments:** | | |
| 1 | Figures 1A and 1B were not sighted in the MSW document which was reviewed | According to PLOS manuscript submission standards, all figures must be uploaded separately via the online submission portal. We did not include figures in the Microsoft word (MSW) because of the instruction, "Do not include figures in the main manuscript file. Each figure must be prepared and submitted as an individual file.". Nevertheless, we included all figure captions and legends in the recommended manuscript section. |
| 2 | Under Findings, p53 metabolism, and conclusions drawn from this study are consistent with previously published work on HIV pathogenesis in relation to p53. What is new? This should be highlighted in the manuscript | The novelty of our study has been highlighted in lines 49 – 53 by replacing the concluding statements “Individuals infected with HIV and occupationally exposed to chemical substances exhibit compromised immunity, elevated oxidative stress, and depressed p53 (loss of tumor suppressive capacity) and bcl2; a convergence promoting the carcinogenic pathway and elevated cancer risk. These findings provide a mechanistic basis of cancer risk and scientific justification for preventive strategies against carcinogenesis in individuals who are HIV-infected”, with “Individuals who are HIV-infected and occupationally exposed to chemicals have a constellation of depressed immunity, elevated oxidative stress, and loss of tumor suppressive functions, which together intensify cancer risk, providing valuable scientific and public health bases for preventive measures in this vulnerable population”. |
| 3 | Authors should clarify if the study is strictly a cross-sectional study. Described methodology has component of case-control design. Check and reconcile this. If it has a mixed- method design explain same | We confirm the choice of a cross-sectional study design for investigating the levels of cancer risk biomarkers in HIV-infected individuals exposed to occupational chemicals, exploring the relationship between apoptotic regulatory markers and DNA oxidative response markers as a measure of cancer risk. The cross-sectional design allows for the simultaneous assessment of HIV status, toxic metal exposure, and cancer risk biomarkers at a single point in time. In this cross-sectional study the exposure (HIV status and toxic metal exposure) and the outcome (levels of cancer risk biomarkers) were measured concurrently. Therefore, the cross-sectional design is adequate for the study objectives. A case-control study may be ideal when there is a specific disease or condition of interest that has already occurred. In our case the study focuses on biomarkers, which are indicators of potential risk rather than established disease states. Our design allows for direct comparisons among the four groups (HIV-positive exposed, HIV-positive unexposed, HIV-negative exposed, and HIV-negative unexposed) to assess the combined and independent effects of HIV infection and toxic metal exposure on cancer risk biomarkers. |
| 4 | Was the questionnaire validated prior to use for the study? Mention same if it was . If it was not, validation will be required | Yes, we confirm that the questionnaire was subjected to pilot testing (validation). The following statements have been added in lines 145 – 148: “Prior to its use in the study, the questionnaire was pilot tested with a small group representative of the study population to ensure clarity and relevance. Additionally, a panel of experts in toxicology and environmental epidemiology reviewed the questionnaire to ensure its validity.” |
| 5 | How did authors ascertain validity of electronic measuring scale? Was the reading taken by one or multiple persons? How did you ascertain inter and intra variation validity. | To improve clarity, the contents, "The weighing scale and stadiometers were calibrated with standard weights and known height measurements, respectively." have been expanded to: "Prior to the study, we used standard weights and known height measurements to calibrate the weighing scale and stadiometers. We performed regular calibration checks throughout the study to maintain measurement accuracy and detect any drifts in the scale's performance. We assessed inter-rater and intra-rater reliability by having multiple research assistants measure height and body weight and comparing the results." Please see lines 176 – 181. |
| 6 | Principle of all chemical based measurements should be explained first before description of procedure | The principle of CD4 enumeration, earlier stated as “This flow cytometry method employs specific monoclonal antibody and fluorescence-activated cell sorter (FACS) analysis,” has been expanded in lines **211–214** to provide more context: “This flow cytometry for CD4 enumeration employs specific monoclonal antibodies tagged with fluorescent dyes to bind CD4+ cells. These cells are then passed through a fluorescence-activated cell sorter (FACS), where they are identified and counted based on their fluorescence intensity.” Furthermore, the principle of ELISA has been described in lines 231 – 234: “First, a specific antibody that is immobilized on a plate binds to the target antigen. Then, a second enzyme-linked antibody is added and binds to it, creating a sandwich. The enzyme substrate reaction produces a detectable signal proportional to the antigen concentration.” |
